# ECM1 is associated with endocrine resistance in ER^+^ breast cancers

**DOI:** 10.1080/19768354.2022.2083235

**Published:** 2022-06-22

**Authors:** Tae Won Lee, Kyung-min Lee

**Affiliations:** a Department of Life Science, College of Natural Sciences, Hanyang University, Seoul, Republic of Korea; b Research Institute for Natural Sciences, Hanyang University, Seoul, Republic of Korea; c Research Institute for Convergence of Basic Sciences, Hanyang University, Seoul, Republic of Korea; d Hanyang Institute of Bioscience and Biotechnology, Hanyang University, Seoul, Republic of Korea

**Keywords:** ECM1, ER^+^ breast cancer, endocrine resistance, luminal breast cancer, Src

## Abstract

Extracellular matrix protein 1 (ECM1) is associated with a poor prognosis of breast cancers. However, the role of ECM1 with endocrine resistance in estrogen receptor-positive (ER^+^) breast cancers has not been elucidated yet. We show that ECM1 promotes endocrine resistance in ER^+^ breast cancers. ECM1 is overexpressed in luminal breast cancer patients compared to the basal type of breast cancer. Significantly, higher expression of ECM1 is associated with poor response to endocrine therapies in luminal B breast cancer patients. We found that ECM1 is upregulated in CAMA1 and MDA-MB-361 cells grown in long-term estrogen-deprived (LTED) conditions. Moreover, the ablation of ECM1 significantly inhibited the proliferation of CAMA1 LTED and MDA-MB-361 LTED cells. Finally, an interrogation of a dataset containing transcriptome and proteome of breast cancer cell lines revealed that the level of *ECM1* mRNA is positively correlated with that of phosphorylated Src. Based on these findings, we strongly suggest that ECM1 significantly contributes to the acquisition of endocrine resistance in ER^+^ breast cancers by the activation of Src.

## Introduction

Breast cancer is the most common malignancy in women worldwide (Harbeck and Gnant 2017). Estrogen receptor-positive (ER^+^) breast cancer is characterized by the expression of estrogen receptors and accounts for about 80% of all breast cancers (Onitilo et al. 2009). ER^+^ breast cancers typically showed relatively mild clinical outcomes compared to estrogen-negative (ER^-^) breast cancers, including HER2-positive and triple-negative breast cancer (Mesa-Eguiagaray et al. 2020).

Endocrine therapies have contributed to the significant decrease in cancer-related mortality in ER^+^ breast cancers (Tremont et al. 2017). However, due to intrinsic or acquired resistance these therapies are effective in a few patients. Recent reports suggested that hot spot mutation of *Estrogen receptor 1* (*ESR1*) can be the primary cause of acquired endocrine resistance (Merenbakh-Lamin et al. 2013; Toy et al. 2013; Fanning et al. 2016). In addition, hyperactivation of phosphatidyl inositol-3-kinase (PI3K) promotes endocrine resistance to systemic depletion of estrogen in the preclinical study (Miller et al. 2010). *Kirsten rat sarcoma viral oncogene homolog* (*KRAS*) mutations also drive endocrine resistance in breast cancer patients (Raimondi et al. 2021). However, many patients do not benefit from endocrine therapies due to resistance to unknown mechanisms.

Extracellular matrix protein 1 (ECM1) is a glycoprotein secreted in the extracellular matrix (Mathieu et al. 1994; Smits et al. 1997) and overexpressed in several cancer types, including breast cancer (Wang et al. 2003). ECM1 has been associated with malignant phenotypes, such as proliferation, migration, invasion, stemness, and therapeutic resistance, in ovarian cancers, HER2 + breast cancers, and triple-negative breast cancers (Lee et al. 2014; Lee, Nam, Oh, Lim, Kim, et al. 2015; Lee, Nam, Oh, Lim, Lee, et al. 2015; Steinhaeuser et al. 2020; Yin et al. 2021). However, the role of ECM1 in ER^+^ breast cancers has not been elucidated.

In this study, we found that *ECM1* is genetically amplified, and its overexpression is significantly associated with a worse prognosis in ER^+^ breast cancers. *ECM1* ablation significantly attenuated the proliferation of LTED cells, a model mimicking acquired resistance to endocrine therapy. Finally, we showed that the expression of *ECM1* in ER^+^ breast cancer is strongly associated with the phosphorylation of Src in ER^+^ breast cancer cells. Our findings propose the implication of ECM1 for endocrine resistance in ER^+^ breast cancers.

## Materials and methods

### Cell culture

CAMA1, MAD-MB-361, and T47D cells were purchased from ATCC in 2019 and grown in DMEM supplemented with 10% fetal bovine serum (FBS), 1% antibiotic-antimycotic solution. Long-term estrogen-deprived (LTED) cells were generated and maintained, as described in the previous report (Miller et al. 2010).

### Western blot analysis

A total of 500,000 cells were seeded in 60-mm dishes. The next day, cells were harvested and then lysed with RIPA lysis and extraction buffer (Cat#89901, ThermoFisher) containing protease inhibitors (Protease Inhibitor Cocktail, Roche) and phosphatase inhibitors (PhosSTOP, Roche) at 4. After 30 min, lysates were centrifugated at 14,000 rpm for 10 min. Supernatants were collected, and then protein concentration was measured with the Pierce BCA protein assay kit (Cat#23227, ThermoFisher). Thirty µg of protein mixed with sample buffer (Cat#NP0007, ThermoFisher) was denatured and then subjected to SDS-PAGE then transferred to a nitrocellulose membrane for western blot analysis. ECM1 (Cat#AF3937) and actin (Cat#4967) antibodies were purchased from R&D systems and Cell Signaling Technology, respectively. The intensities of the bands were quantified using ImageJ (Schneider et al. 2012).

### Gene knockdown using siRNA

Cells were seeded in 6-well plates or 60-mm dishes. The next day, cells were transfected with 20–40 pmole of siRNA targeting ECM1 (Cat#4392420, Assay ID s4441, ThermoFisher) or control siRNA (Cat#4390824, Assay ID s10520, ThermoFisher) using Lipofectamine RNAiMax (Cat# 13778075, Invitrogen) according to the manufacturer’s instruction.

### Cell proliferation assay

After 24 h from the transfection of siRNA, 10,000 cells were seeded in 6-well plates. Cells were trypsinized and then counted every 3 days for 6 days using a Z2 coulter counter analyzer (Beckman coulter).

### Real-time quantitative polymerase chain reaction (RT-qPCR)

RNA was extracted from cells using Maxwell RSC simply RNA Cells Kit, (Promega) according to the manufacturer’s protocol. Complementary DNA was synthesized using the iSCRIPT cDNA synthesis Kit (Cat#1708890, Bio-Rad) and then subjected to PCR with PowerUp^TM^ SYBR^TM^ Green Master Mix (Cat#A25741, ThermoFisher) using a QuantStudio3 Real-Time PCR System (ThermoFisher). *ECM1* (Cat#330001, GeneGlobe ID PPH01460E) and *GAPDH* (Cat#330001, GeneGlobe ID PPH00150F) primers were purchased from Qiagen.

### Statistical analyses

All the data of in vitro studies were obtained from three independent experiments. Data are presented as mean ± SD. Statistical analyses were performed using RStudio and ANOVA. A *P-*value of less than 0.05 was considered statistically significant.

## Results

### ECM1 is overexpressed in luminal breast cancers

**Figure 1.**
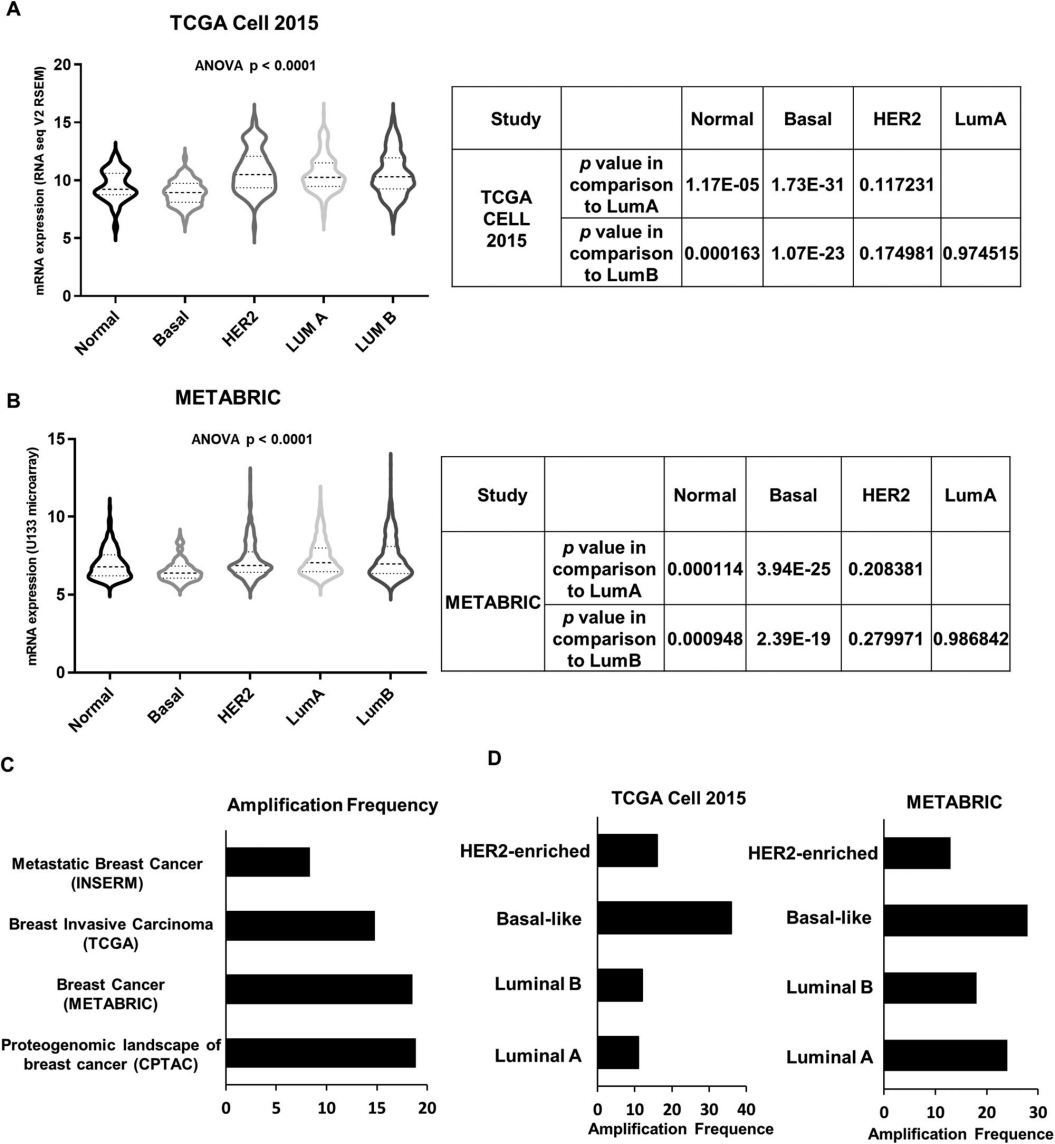
ECM1 is overexpressed in luminal types of breast cancer. (A) Frequency of *ECM1* copy number in CPTAC, METABRIC, TCGA or INSERM breast cancers. (B) Alteration frequency of *ECM1* by PAM50 molecular subtype in TCGA (left panel) and METABRIC (right panel) breast cancers. (C and D) Level of *ECM1* mRNA by PAM50 subtypes in TCGA (C) and METABRIC (D) breast cancers. Tables contain *p* values for the corresponding comparison.

To investigate the expression level of ECM1 in ER^+^ breast cancers, we analyzed the transcriptome data from The Cancer Genome Atlas (TCGA) (Ciriello et al. 2015) and METABRIC (Curtis et al. 2012) breast cancers by subtypes classified by PAM50 (Parker et al. 2009). The analyses revealed that *ECM1* is highly expressed in HER2-enriched and luminal subtypes compared to basal-like and normal-like subtypes in TCGA breast cancers (Figure 1(A)). Consistently, the expression level of *ECM1* is significantly higher in luminal subtypes than in other subtypes in METABRIC breast cancers (Figure 1(B)).

*ECM1* is located in the 1q21 locus that is frequently amplified in breast cancers and associated with worse clinical outcomes (Goh et al. 2017). Thus, we examined the copy number alteration (CNA) of the *ECM1* in ER^+^ breast cancers within datasets available in cBioPortal (Cerami et al. 2012). The rate of *ECM1* copy number amplification was about 19%, 19%, 15%, and 8% in the Clinical Proteomic Tumor Analysis Consortium (CPTAC) (Krug et al. 2020), METABRIC, TCGA, and National Institute of Health and Medical Research (INSERM) (Lefebvre et al. 2016), respectively (Figure 1(C)). Finally, we examined the rate of *ECM1* CNA in each PAM50 subtype of TCGA and MEABRIC breast cancers. In both datasets, approximately 10–20% of luminal types of breast tumors harbored *ECM1* amplification (Figure 1(D)). These suggest that *ECM1* is amplified and consequently overexpressed in breast cancers, including luminal subtypes.

### ECM1 is associated with poor outcomes in luminal B breast cancer

**Figure 2.**
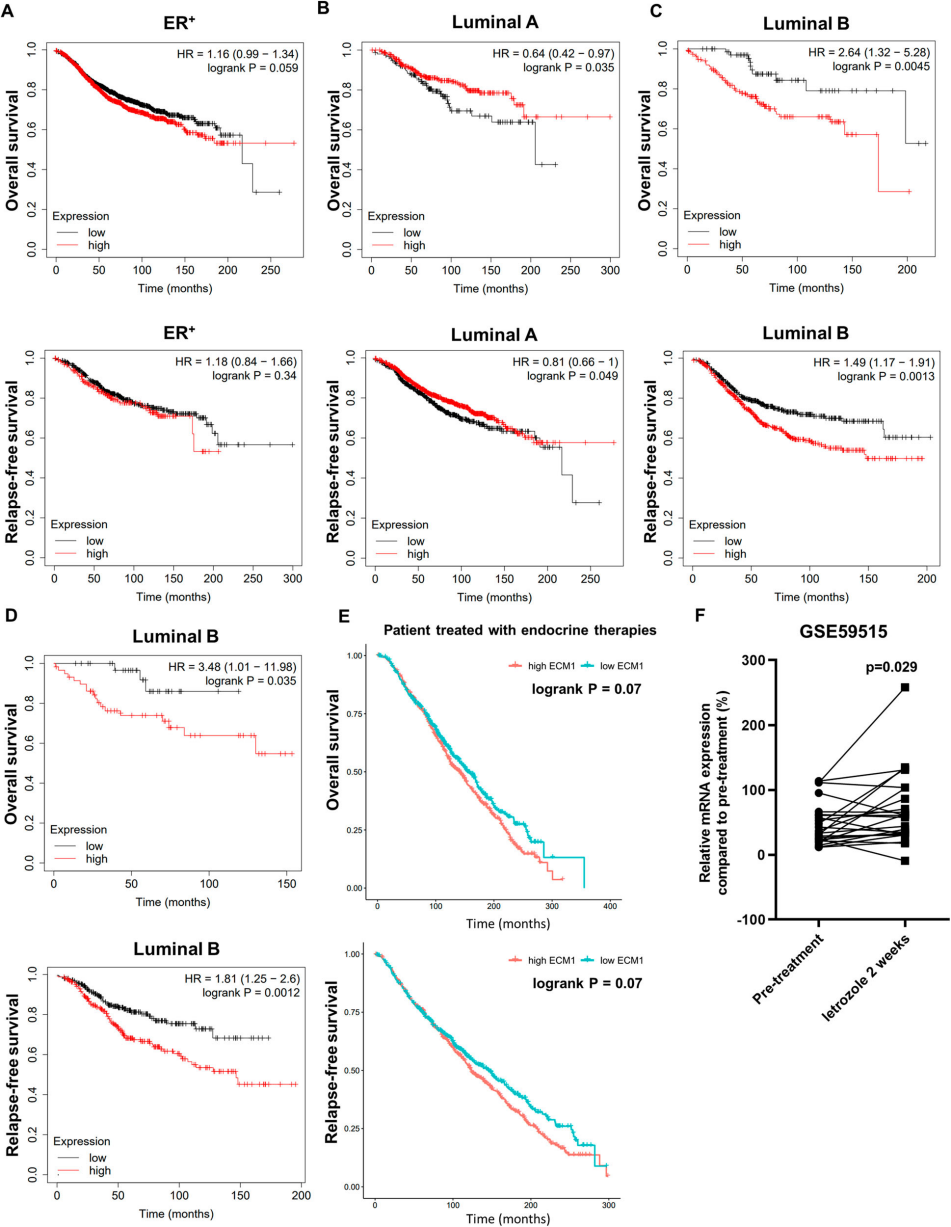
ECM1 is associated with poor outcomes of luminal B-type breast cancers. (A) OS and RFS of ER^+^ breast cancer patients with low or high *ECM1* mRNA levels by the autoselect best cutoff in the Kaplan-Meier Plotter. (B) OS and RFS of luminal A breast cancer patients with low or high *ECM1* mRNA levels by the autoselect best cutoff in the Kaplan-Meier Plotter. (C) OS and RFS of luminal B breast cancer patients with low or high *ECM1* mRNA levels by the autoselect best cutoff in the Kaplan-Meier Plotter. (D) OS and RFS of luminal B breast cancer patients who were treated with hormone therapies, with low or high *ECM1* mRNA levels. (E), OS and RFS of METABRIC breast cancer patients with low or high *ECM1* mRNA levels. (F) Relative expression of *ECM1* in post-treatment ER^+^ tumors compared to pre-treatment tumors.

Next, we investigated whether high expression of ECM1 is associated with a poor prognosis of luminal breast cancers. Survival analyses using the Kaplan-Meier plotter (Lanczky and Gyorffy 2021) showed that the level of *ECM1* expression is not prognostic in the whole group of ER^+^ breast cancer patients (Figure 2(A)). However, subgroup analyses revealed that higher expression of *ECM1* is significantly associated with shorter overall survival (OS) and relapse-free survival (RFS) in luminal B type but not in luminal A-type (Figure 2(B and C)). Considering the relatively higher recurrence rate following endocrine therapy in luminal B type compared to luminal A type (Dowsett et al. 2013), these may suggest a role of ECM1 in resistance to endocrine therapies. To gain more insight into this notion, we assessed whether the expression level of *ECM1* is prognostic in ER^+^ breast cancers treated with endocrine therapy. Interestingly, high expression of *ECM1* was strongly associated with short RFS and OS in luminal B breast cancer patients following endocrine therapy (Figure 2(D)). In addition, we found that high expression of *ECM1* is associated with worse outcomes in METABRIC ER^+^ breast cancers treated with endocrine therapy (Figure 2(E)). Finally, an interrogation of the GSE59515 dataset from a study with comparative gene expression profiling of ER^+^ breast tumors receiving neoadjuvant letrozole treatment for 2 weeks (Selli et al. 2016) showed that the expression of *ECM1* is increased in post-treatment tumors compared to pre-treatment tumors (Figure 2(F)). These data imply that ECM1 overexpression contributes to the acquisition of resistance to endocrine therapy in luminal B breast cancers.

### ECM1 is highly expressed in cell line models that acquire endocrine resistance

**Figure 3.**
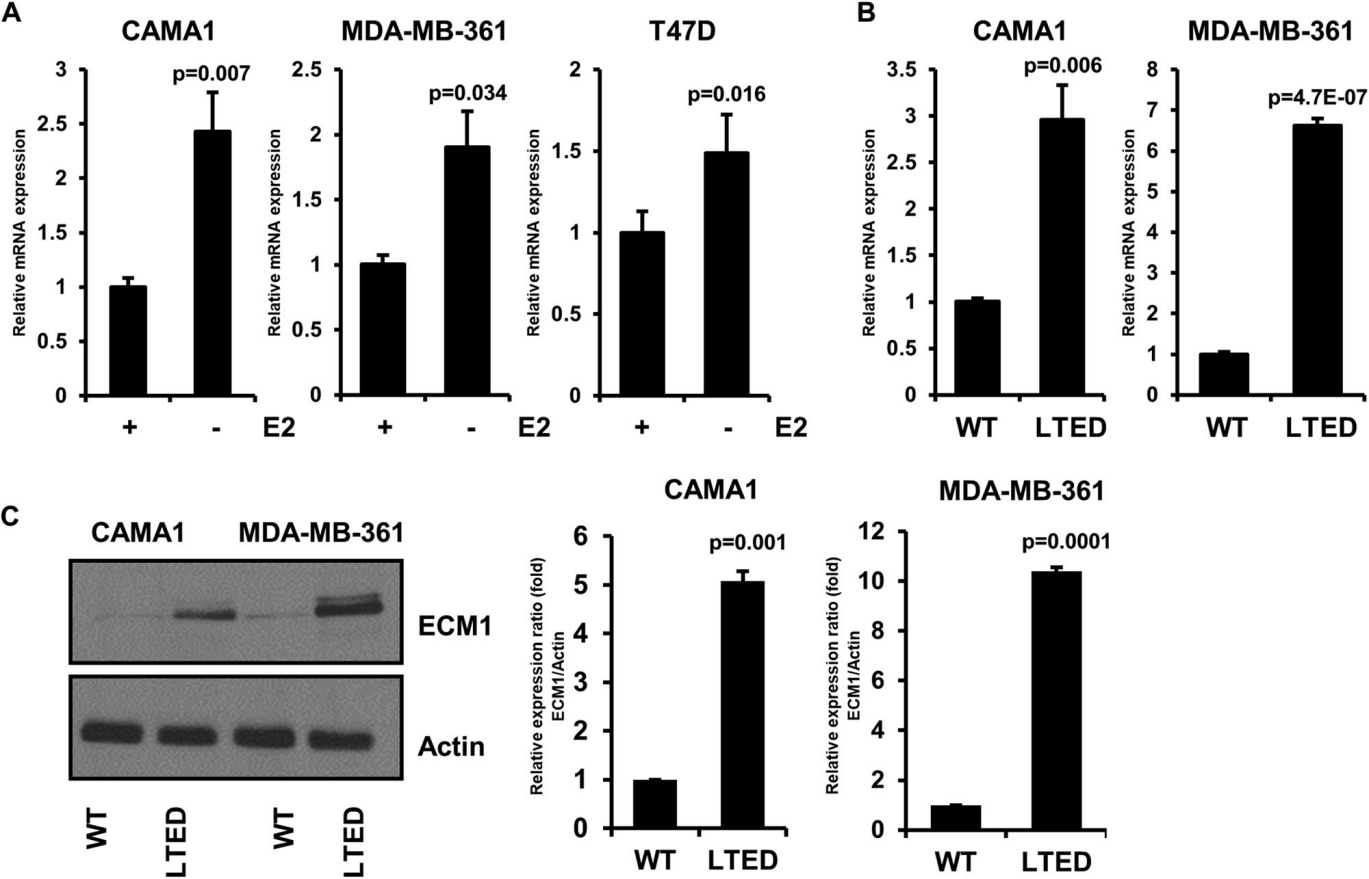
ECM1 is highly expressed in ER^+^ breast cancer cells with acquired endocrine resistance. (A) Complementary DNA (cDNA) was synthesized by mRNA extracted from CAMA1, MDA-MB-361, and T47D cells grown ± 1 nM E2 for 7 days. cDNA was subjected to real-time quantitative PCR (RT-qPCR) B and C, Levels of *ECM1* mRNA (B) and protein (C) in CAMA1 LTED and MDA-MB-361 LTED cells in the absence of E2.

To validate the induced ECM1 following endocrine therapy, we next determined the expression of ECM1 in ER^+^ breast cancer cells grown in the estrogen-deprived condition. Consistent with clinical samples, the short-term estrogen deprivation induced *ECM1* expression in CAMA1, MDA-MB-361, and T47D cells, all of which are classified as ER^+^ breast cancer cell lines (Figure 3(A)). Next, we employed CAMA1 and MDA-MB-361 cells grown in the long-term estrogen deprivation (LTED) condition, which are representative models that mimic the acquired resistance to estrogen depletion (Miller et al. 2010). The expression of ECM1 was also elevated in CAMA1 LTED and MDA-MB-361 LTED cells compared to the corresponding wild-type cells (Figure 3(B)). Furthermore, we validated that CAMA1 LTED and MDA-MB-361 LTED cells overexpress ECM1 in protein levels (Figure 3(C)).

### ECM1 ablation inhibits the proliferation of endocrine-resistance ER^+^ breast cancer cells

**Figure 4.**
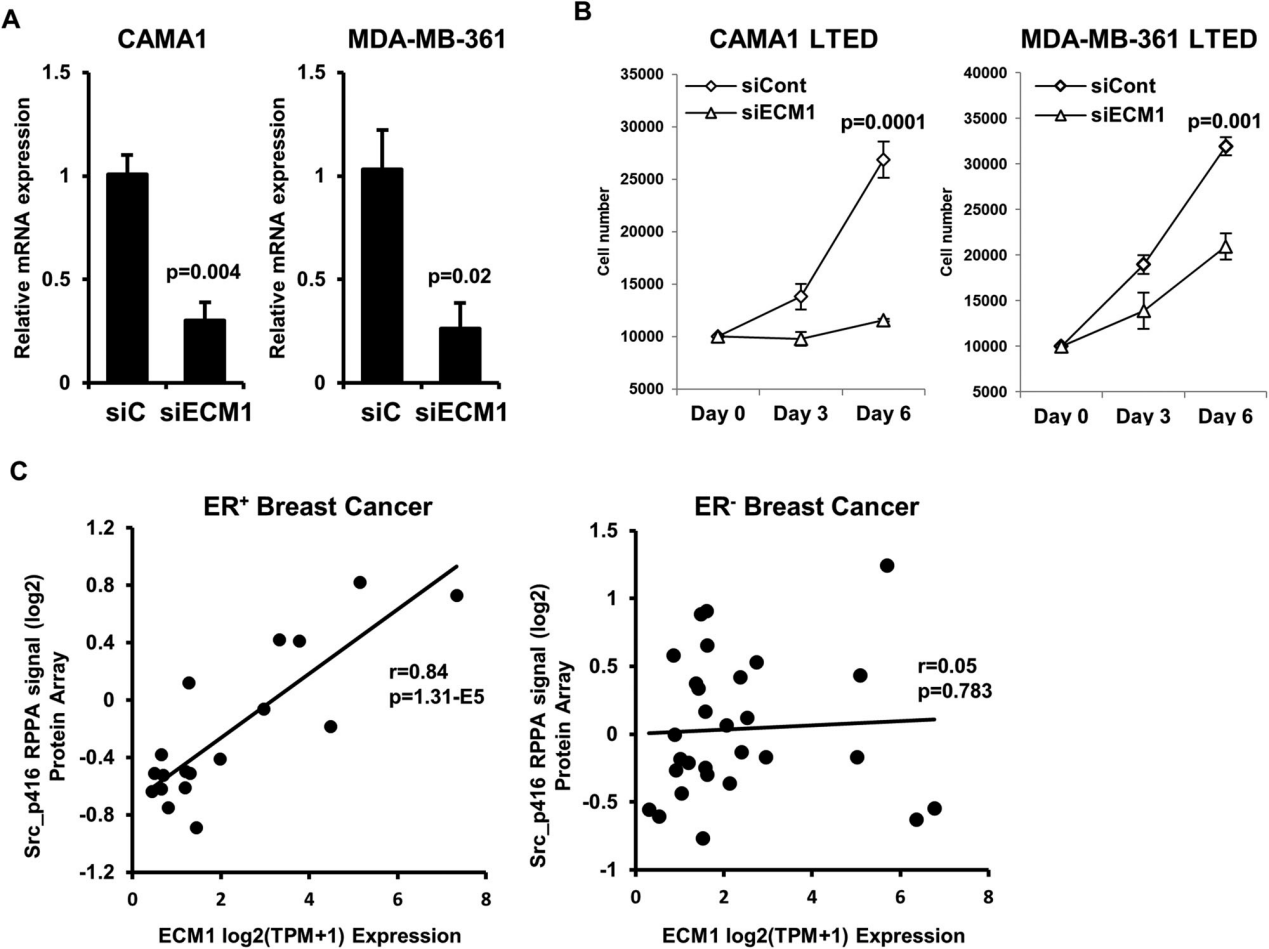
ECM1 ablation inhibits the proliferation of endocrine-resistant ER^+^ breast cancer cells. (A) mRNA extracted from CAMA1 LTED and MDA-MB-361 LTED cells, transfected with either control siRNA or *ECM1* siRNA for 48 h, were subjected to RT-qPCR. (B) the number of cells described in A was counted every 3 days for 6 days. (C) Correlation between levels of *ECM1* expression and levels of phosphorylated Src at Y416 residue was assessed in ER^+^ breast cancer cell lines (left panel) and ER- breast cancer cell lines (right panel) using the DepMap dataset.

We next assessed the functional role of ECM1 in ER^+^ breast cancer cells that acquire endocrine resistance. Ablation of ECM1 using a small-interference RNA (siRNA) targeting *ECM1* transcripts resulted in the significant reduction of proliferation of CAMA1 LTED and MDA-MB-361 LTED cells (Figure 4(A and B)). To reason molecular mechanisms(s) by which ECM1 is required for the growth of endocrine-resistant ER^+^ breast cancer cells, we interrogated Cancer Dependency Map (DepMap) database (Ghandi et al. 2019), and then found that the level of *ECM1* mRNA is positively correlated with that of Src phosphorylation at the tyrosine 416 (Y416) residue in ER^+^ breast cancer cell lines, but not in ER^-^ breast cancer cell lines (Figure 4(C)). The phosphorylation of Src at Y416 residue has been shown as a surrogate marker representing its activation that eventually increases cell proliferation in various cancer types (Roskoski 2004). Furthermore, the activation of Src promotes endocrine resistance in ER^+^ breast cancer cells (Hiscox et al. 2006; Guest et al. 2016). Collectively, these imply that ECM1 contributes to the activation of Src, which promotes cell proliferation and endocrine resistance in ER^+^ breast cancer cells.

## Discussion

Several genetic alterations have been shown as a driver of de novo and acquired resistance to endocrine therapy (Hanker et al. 2020). For instance, *Erb-B2 receptor Tyrosine Kinase 2* (*ERBB2*) activating mutations are found in 5% of endocrine-resistant metastatic breast cancers (Razavi et al. 2018). Copy number amplification of *ERBB2* reduces the sensitivity of endocrine therapy by the activation of mitogen-activated protein kinase (Kurokawa et al. 2000). In addition, our recent work revealed that copy number amplification of *Proline-rich 11* (*PRR11*) promotes resistance to endocrine therapy in ER^+^ breast cancers (Lee et al. 2020). Herein, we identified that *ECM1* that is genetically amplified in breast cancers is crucial for the survival of ER^+^ breast cancer cells with the acquired endocrine resistance.

Mechanistically, ECM1 has been associated with the aberrant activation of several kinases. ECM1 promotes a Warburg effect-like metabolic phenotype (Lee, Nam, Oh, Lim, Lee, et al. 2015), cell proliferation, and trastuzumab resistance (Lee et al. 2014) by the activation of the EGFR pathway in breast cancers. In addition, ECM1 promotes gastric cancer metastasis by the activation of FAK (Focal adhesion kinase) (Gan et al. 2018). More recently, ECM1 secretory isoform binds to integrin αxβ2, which activates the AKT/FAK/Rho pathway in ovarian cancers (Yin et al. 2021). We showed that the level of *ECM1* mRNA is strongly and positively correlated with that of phosphorylated Src at Y416 residue, a surrogate marker for its activation, in ER^+^ breast cancer cells, suggesting that ECM1 may promote endocrine resistance by the activation of Src. Src, a non-receptor tyrosine kinase, contributes to tumorigenesis in various cancer types (Abula et al. 2021) and, more specifically, promotes endocrine resistance in breast cancers (Hiscox et al. 2009; Morgan et al. 2009; Vallabhaneni et al. 2011). Moreover, the role of ECM1 in the activation of Src by FAK has been identified in ovarian and gastric cancers (Yin et al. 2021) (Gan et al. 2018). Altogether, these previous findings further support our notion regarding the activation of Src by ECM1.

The present study showed that overexpression of *ECM1* is associated with worse clinical outcomes and endocrine resistance to ER^+^ breast cancer. Analyses of gene expression profiles from clinical specimens proposed ECM1 as a biomarker indicative of resistance to endocrine therapy. Mechanistically, the activation of Src is predicted as a driver of ECM1-mediated endocrine resistance; however, the precise molecular mechanism by which ECM1 induces phosphorylation of Src remains unclear. Thus, a follow-up study pursuing the molecular mechanism will be required soon.

## Data Availability

The data that support the findings of this study are openly available in cBioPortal at DOI: 10.1126/scisignal.2004088, DepMap portal at https://depmap.org/portal/. mRNA expression data in TCGA (Ciriello et al. 2015) and METABRIC dataset (Pereira et al. 2016) were also downloaded from cBioPortal. Kaplan-Meier survival plots were generated from the Kaplan-Meier plotter (https://kmplot.com/analysis/). Correlation scatter plots and bar plots were generated from RStudio, version 4.1.1 (RStudio Team (2020). RStudio: Integrated Development for R. RStudio, PBC, Boston (http://www.rstudio.com/)).
